# Cancer Risk and *GSTM1* and *GSTT1* Polymorphisms: Hansteen et al. Respond

**DOI:** 10.1289/ehp.0900829R

**Published:** 2009-07

**Authors:** Inger-Lise Hansteen, Anna Maria Rossi, Roberto Barale, Lisbeth E. Knudsen, Hannu Norppa, Stefano Bonassi

**Affiliations:** Department of Laboratory Medicine, Section of Medical Genetics, Telemark Hospital, Skien, Norway; Department of Biology, Pisa University, Pisa, Italy; Environmental Health, Institute of Public Health, University of Copenhagen, Copenhagen, Denmark; New Technologies and Risks, Work Environment Development, Finnish Institute of Occupational Health, Helsinki, Finland; Unit of Molecular Epidemiology, National Cancer Research Institute, Genoa, Italy, E-mail: stefano.bonassi@istge.it

We thank Cetta et al. for the interesting comments regarding our article (Rossi et al. 2009). In their letter they address two main issues. The first refers to the role of genetic polymorphisms in the causal relationships between exposure to carcinogens and cancer occurrence. The second is more conceptual and criticizes the evolution of association studies, claiming a decreased attention to pathogenetic mechanisms in favor of an indiscriminate increase of the study size, with a consequent lack of biological plausibility.

We agree that these are important issues. We have addressed the problem of inherited predisposition for DNA damage from a different angle, namely using the frequency of chromosomal aberration (CA) as a response indicator for occupational and environmental exposure to genotoxic agents. An increase in CA level in exposed individuals compared with controls has been documented since the 1990s (Nordic Study Group 1990). The conceptual basis for using this assay has been the hypothesis that the extent of genetic damage in peripheral lymphocytes reflects critical events for the carcinogenic process in target tissues.

The key issue—whether the association with cancer risk is attributable to exposure to carcinogenic agents or reflects inherited susceptibility and accumulated damages—was addressed with a nested case–control study on incident and deceased cancer cases in the Nordic and Italian cohorts (Bonassi et al. 2000). The main findings of that study indicated an increase in cancer risk for subjects with high CA levels compared with those with low levels. This increase was independent of exposure history, as further verified in follow-up studies (Bonassi et al. 2008; Hagmar et al. 2004).

In all these studies, cancer has been studied as one entity. This summarization was mostly due to statistical needs, although the very early occurrence of chromosome damage in the carcinogenic pathway of most solid cancers provided a valuable rationale (Mitelman et al. 2004). A further reason for summarizing data by cancer type was that damages were measured in surrogate tissues and not in the target, providing only an indirect measure of cancer-related events. However, studying the cancer site in relation to CA frequency was a major interest of our group, because different types of cancers have different pathogenetic models. In our recent article (Rossi et al. 2009), we grouped cancer types into three groups, and we showed for all of these groups that subjects with high levels of CAs are more susceptible to developing cancer than are subjects with low or medium levels of CAs; this indicates that CA is an inherited susceptibility marker for cancer regardless of cancer type.

The beginning of Cetta et al.’s letter is misleading. The statement from our article (Rossi et al. 2009) that “*GSTM1* [gluta-thione *S*-transferase M1] and *GSTT1* [gluta-thione *S*-transferase theta 1]polymorphisms [as all individual polymorphisms] . . . are not expected to have a dramatic influence on baseline CA [chromosomal aberration] or overall cancer risk” is not a conclusion of the study, but describes the conclusions of the extensive literature supporting this evidence (Hirschhorn 2009). We agree that it is important to examine the cause of different types of cancer and the role(s) of the different modifying enzymes, including *GSTM1* and *GSTT1*. However, the present study was designed to evaluate a possible modifying effect of *GSTM1* and *GSTT1* on the cancer predictivity of CA (indicating individual susceptibility to developing cancer). Our main concern was identify individuals more susceptible to damage from known genotoxic exposure. Because only *GSTM1* and *GSTT1* polymorphisms have been extensively evaluated in human surveillance studies, we tested only these genotypes. Within the consortium of studies included in this project (Bonassi et al. 2008), further follow-up studies to differentiate cancer types or include other genotypes are possible, providing adequate financial support.

The issue raised by Cetta et al. of decreased attention to pathogenetic mechanisms in favor of larger studies, with a consequent lack of biological plausibility, is only partially correct. Actually, in association studies that link a genetic polymorphism to the effect of exposure or to the risk of cancer, the lack of specificity is the main reason for failure. Another reason for their failure is small study size, which generates meaningless and often contrasting results. The conflict noted by Cetta et al. is apparent because, as demonstrated by the success of genome-wide association studies, the need of reaching a proper statistical power is as important as studying a genetic polymorphism in a specific pathway.
